# A comprehensive study of the basic formulation of supersaturated self-nanoemulsifying drug delivery systems (SNEDDS) of albendazolum

**DOI:** 10.1080/10717544.2021.1986601

**Published:** 2021-10-06

**Authors:** Hani Alothaid, Mohammed S. Aldughaim, Azeez Oriyomi Yusuf, Umama Yezdani, Alaa Alhazmi, Mahmoud M. Habibullah, Mohammad Gayoor Khan

**Affiliations:** a Department of Basic Sciences, Faculty of Applied Medical Sciences, Al Baha University, Al-Baha, Saudi Arabia; b Research Center, King Fahad Medical City, Riyadh, Saudi Arabia; c School of Biotechnology, Dublin City University, Dublin, Ireland; d Department of Pharmacy Practice, MRM College of Pharmacy, Hyderabad, India; e Medical Laboratory Technology Department, Faculty of Applied Medical Sciences, Jazan University, Jazan, Saudi Arabia; f SMIRES for Consultation in Specialized Medical Laboratories, Jazan University, Jazan, Saudi Arabia; g Department of Pharmacovigilance, UCB, Bioclinica, Mysore, India

**Keywords:** Nanoemulsifying drug delivery system, albendazole, SNEDDS, S-SNEDDS, SS-SNEDDS, microcrystalline

## Abstract

Albendazolum (ABZ) is a BCS class II drug. It has challenging biopharmaceutical properties, which include poor solubility and dissolution rate. These properties have laid the ground for developing a supersaturated self-nanoemulsifying drug delivery system (S-SNEDDS) to form oil-in-water nanoemulsion *in situ* to improve the oral bioavailability of ABZ. Based on the ABZ solubility, emulsifying ability, and stability after dispersion in an aqueous phase, an optimal self-nanoemulsifying drug delivery system (SNEDDS) consisting of oleic acid, Tween^®^ 20, and PEG 600 (X:Y:Z, w/w) was identified, having 10% (w/w) hydroxypropyl methylcellulose (HPMC) E15 lv as its precipitation inhibitor. The optimized system possessed a small mean globule size value (89.2 nm), good dispersion properties (polydispersity index (PDI): 0.278), and preserved the supersaturated state of ABZ. S-SNEDDS was transformed into solid supersaturated self-nanoemulsifying drug delivery systems (SS-SNEDDS) using microcrystalline cellulose as a solid material. The developed S-SNEDDS were characterized for globule size, pH, turbidity, differential scanning calorimetry (DSC), scanning electron microscopy (SEM), and flow properties. The data obtained from the results suggest that this S-SNEDDS formulation can enhance the solubility and oral bioavailability of ABZ for appropriate clinical application.

## Introduction

More than 70% of commercially available drugs are known to have poor aqueous solubility, which significantly affects their bioavailability (Khadka et al., 2014). To circumvent this problem, hydrophobic drugs are often encapsulated within lipid systems, such as liposomes and micelles, which have a bilayer of hydrophilic surfaces that interacts with the aqueous environment, and hydrophobic core, which holds the hydrophobic drugs. Several successful oral pharmaceutical products have been marketed as lipidic systems; notably, cyclosporine A, ritonavir, sanquinavir, and albendazole (ABZ) (Pouton, 2000; Grove et al., 2006). Consequently, there is considerable interest in the potential of lipid formulation for oral administration, with emphasis on liquid self-emulsifying drug delivery systems (SEDDS). SEDDS are comprised of a mixture of surfactants, oils, and solvents, with the ability to re-arrange within an aqueous environment to improve oral absorption. However, the commercial application of this technology is still limited because the efficiency of oral absorption of SEDDS drugs is dependent on factors such as oil to surfactant ratio, surfactant concentration, the polarity of the emulsion, and the size and charge of droplets (Singh et al., 2017; Ghosh et al., 2020; Khursheed et al., 2020; Pandey et al., 2020; Kumar et al., 2021).

Liquid SEDDS solidification has been developed to address the deficiencies of conventional liquid SEDDS (Chavan et al., 2015). Solid SEDDS possesses enhancement solubility and bioavailability in addition to properties of solid dosage formulations such as relatively lower production cost, better stability, the convenience of process control, and reproducibility (Mullertz et al., 2010; Kang et al., 2012; Tan et al., 2013). Many recent studies on the development of solid SEDDS, in which lipids and surfactants were adsorbed onto the solid carrier, have been reported in the literature (Balakrishnan et al., 2009; Beg et al., 2012; Kazi et al., 2017; Shahba et al., 2017).

Self-nanoemulsifying drug delivery system (SNEDDS) pre-concentrates are isotropic mixtures of oil, a surfactant, co-solvent, and drug, which readily disperse in aqueous environments upon mild agitation to generate ultrafine nanoemulsions. The rationale for using SNEDDS for the delivery of poorly water-soluble drugs is that the drug in such pre-concentrates is presented in solution (Mohsin et al., 2009; Kazi et al., 2020). Hence, the required dissolution step for solid and crystalline compounds is avoided. Additionally, upon dispersion and subsequent digestion of SNEDDS, a variety of colloidal structures that are smaller than the parent SNEDDS are formed, thus facilitating drug absorption (Fatouros et al., 2007; Chakraborty et al., 2009; Fatouros et al., 2009). While some attempts have been made to systematically investigate the properties of solid carriers on the drug release profile from SNEDDS by previous studies (Rajesh et al., 2018; Sharma et al., 2018; Ghosh et al., 2020), the impact of physicochemical properties of these drug carriers has not been widely investigated. In the present study, the impact of certain physicochemical properties of solid carriers on drug release from solid SNEDDS is evaluated.

Albendazole is a BCS class II drug with challenging biopharmaceutical properties such as poor solubility and slow dissolution rate. These properties have lain the ground for the development and optimization of the lipid-based formulation of ABZ. Supersaturated SEDDS are thermodynamically unstable and tend to precipitate before absorption by interfering with drug nucleation and crystal growth or by leading to changes in aqueous medium properties. This results in compromised bioavailability. In this study, we developed optimized supersaturated SNEDDS (S-SNEDDS) of ABZ, a class of SNEDDS containing very high concentrations of the drug via optimal response surface design. The optimized S-SNEDDS have higher solubility and better physical stability than previously reported S-SNEDDS. Solid SSNEDDS (SS-SNEDDS) were prepared by adsorbing S-SNEDDS on solid carriers and then evaluated for drug release. We selected microcrystalline cellulose as a solid carrier based on its physicochemical properties, including surface area porosity, hydrophobicity, and hydrophilicity. Microcrystalline cellulose has been selected in this study because of its wide application in direct compression as well as its excellent property as a strong binder, lubricant, filler, and anti-adherent (Thoorens et al., 2014).

## Materials and methods

### Materials

The ABZ (99.0% purity) was received as a generous gift from Zim Laboratories (Nagpur, India). Tween 20v, PEG 600 was obtained from Sigma-Aldrich (St. Louis, MO). Oleic acid and hydroxypropyl methylcellulose (HPMC) were obtained from Rankem and Colorcon Asia Pvt. Ltd. (Verna, India), respectively. MCC, Acros Organics (Geel, Belgium), potassium dihydrogen phosphate, and sodium hydroxide were purchased from Fisher Scientific (Waltham, CA). All other reagents were of analytical grade and used without further purification.

### Determination of ABZ solubility in the oil, surfactant, and co-surfactant fractions

The solubility tests were carried out on various oils, surfactants, and co-surfactants. The solubility determination method previously used by Meena et al. (2012) was employed. Excess ABZ was added to a volume of 1 g of the tested oil, surfactants, and co-surfactants, and placed in well-stoppered vials. The vials were agitated for 72 h (specified after determination of the equilibrium time beyond which the solubility is stable) at 37 ± 0.05 °C in a constant temperature water bath until equilibrium was attained. Subsequently, the vial’s contents were centrifuged at 4000 rpm for 15 min using an ultracentrifuge to separate the insoluble ABZ. After being diluted with the solvent mixture (methanol and 1 N HCl with 7:3), the supernatants were filtered through a cellulose filter (0.022 μm), and the ultraviolet absorbance of the filtrates was measured using the tested oil diluted with the solvent mixture as a blank to be 248 nm using a UV spectrophotometer. The materials used in the study included sunflower oil, Captex 300, PEG 300. PEG 400, soya oil, olive oil, crodamol oil, corn oil, Lauroglycol FCC, Cremophor EL, Solutol HS15, Labrafac lipophile WL1349, Capmul PG8, SPAN 20, Captex 355, Tween 80, Acrysol K150, Labrasol, Cremophor RH40, Acrysol K140, Maisine, Sesame oil, Capmul MCM, Capryol PGMC, and propylene glycol. Results from each experiment were expressed as values in μg/mL.

### Construction of ternary phase diagrams

Ternary phase diagrams using the selected oil (oleic acid), surfactant (tween 20), and co-surfactants (PEG 600) were constructed to identify the specific locations at which self-emulsification occurred by dilution using previously published method (Elkasabgy, 2014). Ternary phase diagrams show the optimum ingredients and ratios of these ingredients in mixtures used for the preparation of a stable SNEDDS. Ternary mixtures were prepared using different combinations of the concentrations of the components, after which a ternary phase diagram was constructed for each mixture. Self-emulsifying ability of each point on the phase diagram was tested by diluting one gram of the corresponding ternary mixture up to 10 mL with distilled water in a capped vial, which was then magnetically stirred at a rotation speed of 125 rpm at 37 °C for 3 min. Phase separation of the diluted mixtures was then evaluated visually. Dispersions with a clear or slight bluish appearance were considered to be in the nanoemulsion region of the diagram.

### Characterization of prepared SNEDDS

In terms of globule size analysis, the self-emulsifying system with the larger nanoemulsion region (oil phase – oleic acid, surfactant – tween 20, co-surfactant – polyethylene glycol (PEG)) was chosen for further evaluation. Different concentrations of its components were prepared, as shown in Table 1. One gram of the prepared self-emulsifying system was introduced into 10 mL of deionized water or phosphate buffer. pH 6.8 was measured after dilution, then magnetic stirring occurred at a rotation speed 125 rpm at 37 °C to mix the diluted system for 3 min. The nano region was selected by briefly centrifuging the suspension at 800×*g* for 10 min at 100 °C to sediment the microvesicles that might have formed. A Particle Size Analyzer (Horiba Scientific SZ-100, Kyoto, Japan) was used to measure both the mean globular size and polydispersity index (PDI) of the diluted SNEDDS.

**Table 1.** Composition of selected SNEDDS.

S. no.	Oil (oleic acid) (%v/v)	Surfactant (tween 20) (%v/v)	Co-surfactant (PEG 600) (%v/v)	Remark
1	5	30	65	Clear
2	5	45	50	Clear
3	5	55	40	Clear
4	5	64	31	Clear
5	5	65	30	Clear
6	5	75	20	Clear
7	5	85	10	Clear
8	10	10	80	Turbid
9	10	20	70	Turbid
10	10	60	30	Turbid
11	10	30	60	Bluish
12	10	40	50	Turbid
13	10	25	65	Turbid
14	10	35	55	Bluish

### Determination of ABZ saturated solubility in the selected SNEDDS

Excess ABZ was added to keep the glass vials containing 1 g of selected optimized SNEDDS preconcentrate (composed of 5% oleic acid, 65% tween 20, 30% PEG) airtight. The suspension was incubated at 30 ± 0.5 °C in a water bath for 72 h to attain equilibrium (Kang et al., 2012). The suspension was then centrifuged for 15 min at 4000 rpm, after three days using an ultracentrifuge to eliminate the undissolved ABZ. This was then followed by filtration of the supernatant with a 0.22 µm membrane filter. After the desired dilution with solvent (methanol and 1 N HCl with 7:3) was achieved, the ultraviolet absorbance of the filtrate was measured using a UV spectrophotometer at 248 nm. Blanks of plain SNEDDS not containing any drugs diluted in the same ratio were used to cancel interference from the SNEDDS preconcentrate. The experiment was repeated thrice, and the average drug saturated solubility in mg/mL of the selected SNEDDS was calculated.

### Preparation of supersaturated SNEDDS

S-SNEDDS preparation was done by dispersing different amounts of HPMC E 15 lv, a precipitation inhibitor into the SNEDDS system. The mixtures were then bath sonicated for 10 min for a uniform HPMC dispersion to be formed. Afterwards, different amounts of the prepared drugs were added to this HPMC dispersion, which was sonicated for another 10 min to ensure good dispersion of the drugs in the system. HPMC E15 lv was added at two different concentrations − 5% and 10%, (w/v). Following this, 50% (s-50%) and 100% (s-100%) drug loads were investigated for their saturated solubility in SNEDDS pre-concentrate. Drug-loaded preparations of either with s-50% or s-100%, without HPMC E15 lv were prepared to evaluate the effect of the precipitation inhibitor or parachute on SNEDDS behavior. Details of the composition of the S-SNEDDS system preparations are shown in Table 2.

**Table 2.** Composition of the S-SNEDDS.

Formulation code	S % (w/v)	HPMC E15 lv % (w/v)
A	50	0
A1	50	5
A2	50	10
B	100	0
B1	100	5
B2	100	10

### Characterization of the prepared S-SNEDDS formulation

#### Globule size analysis

Values of the globule size and PDI were determined for the different S-SNEDDS formulations to investigate the effect of the added drug loads and HPMC E15 lv on their properties as previously reported (Elkasabgy, 2014). The mean globular size and PDI of the diluted SNEDDS were measured using dynamic light scattering of the hydrodynamic radius of the nanoparticles in the Particle Size Analyzer (Horiba Scientific SZ-100, Kyoto, Japan).

#### pH determination

To investigate any ionization of lipid hydrogen or aqueous content of samples, the pH of the prepared SNEDDS pre-concentrate was determined using a digital pH meter.

#### Turbidity measurement

Turbidity measurements were used to identify the efficiency of self-emulsification by establishing whether the dispersion reached equilibrium rapidly and in a reproducible time. These measurements were taken using UV visual spectroscopy (i.e. absorbance of suitably diluted aqueous dispersion at 248 nm).

#### Preparation of the solid supersaturated self-nanoemulsifying drug delivery system

Optimized S-SNEDDS was solidified by adsorbing it on 1 g of the solid carrier. The S-SNEDDS was added to the solid carrier in a drop-wise manner with continuous mixing with a mortar and pestle to obtain a homogeneous free-flowing powder of SS-SNEDDS.

### Characterization of prepared SS-SNEDDS formulation

#### Bulk density

The bulk density of a material is the ratio of the mass to the volume (including the interparticulate void volume) of an untapped powder sample. Two grams of powder blend was introduced to a 10 mL measuring cylinder. The initial volume was then noted (Chaudhari et al., 2013).

Bulk density = weight of powder/bulk volume.

#### Tapped density

The tapped density was measured by mechanically tapping a graduated cylinder containing the sample until almost no further volume change was observed. The powder was added to a measuring cylinder, after which the device was mechanically tapped. After 500 taps, the volume was measured (Sousa e Silva et al., 2013).

Tapped density = weight of powder/tapped volume.

#### Hausner’s ratio

Hausner’s ratio is the ratio of bulk volume to tapped volume or tapped density to bulk density (Shah et al., 2008).

Hausner ratio = tapped density/bulk density.

#### Angle of repose

The angle of repose of the powder blend was evaluated using the funnel method. The accurate weight of the powder blend was measured in a funnel, whose height was adjusted such that the tip of the funnel touched the apex of the powder surface. The diameter of the powder cone was measured and angle of repose was calculated (Shah et al., 2008).

Tan θ=h/r

where *θ* is the angle of repose, *h* is the height of the powder cone, and *r* is the radius of the powder cone.

#### Compressibility index

Compressibility is indirectly associated with the relative flow rate and particle size distribution of the powder. Tapped (td) and apparent bulk density (bd) measurements were used to estimate the compressibility of the nanoparticles, as previously described by Shah et al. (2008) using the equation below and classification in Table 3.

Formula=[(tapped density−bulk density)×100]/tapped density.

**Table 3.** Hausner’s ratio range, angle of repose range, and compressibility index range.

Hausner’s ratio range	Angle of repose range	Compressibility index	Category
1.2–1.3	25–30	5–15	Excellent
1.3–1.4	30–35	12–16	Good
1.4–1.5	35–40	18–21	Fair
1.5–1.6	40–45	23–35	Poor
	45–50	35–38	Very poor
		More than 40	Extremely poor

#### Differential scanning calorimetry (DSC)

DSC analysis of the SNEDDS was conducted on a DSC Polyma 214 that is equipped with cooling system and operating with universal analysis 214 software version NETZSCH. Dry nitrogen was used to purge the sample cell at a flow rate of 80 mL/min. The DSC instrument was calibrated heat flow and for temperature using indium standard of high purity. Accurately weighed samples (1–3 mg) were scanned at a heating rate of 10 °C/min.

#### Scanning electron microscopy (SEM)

Scanning electron microscopy (Sigma 300, Zeiss, Oberkochen, Germany) was employed for evaluating the surface morphology of SS-SNEDDS. Lyophilized powder was deposited on a glass coverslip, which was previously adhered using carbon tape attached to a metallic stub. This was then air-dried and further metalized with gold coating using a vacuum sputter. This sample was then analyzed using SEM (Sigma 300, Zeiss, Oberkochen, Germany).

## Results and discussion

### Determination of ABZ solubility in the investigated oils, surfactants, and co-surfactants

Various oils, surfactants, and co-surfactants were used in this study. The results of solubility studies of ABZ in various oils, surfactants, and co-surfactants are presented in Table 4. It is evident from the data obtained that ABZ does not have good solubility with any of the excipients used. The highest solubility of ABZ at 734.20 µg g^−1^ was found in oleic acid. This finding agrees with the findings of Larsen et al. (2012), where different SNEDDS formulations of cinnarizine, a poorly water-soluble substance, were prepared. The authors found oleic acid to be the most superior excipient in comparison with sesame oil and Cremophor RH40. Oleic acid is known to have variable pKa depending on whether it is ionized or protonated, thus allowing oleic acid to act like an oil or a surfactant (Patel et al., 2013). The properties of oleic acid vary with pH. For instance, the p*K*_a_ of oleic acid in water is 9.85, and it decreases to 6.5 when fatty acid is added to a mixture of bile acid and phospholipid micelles. However, the properties of ionized and protonated oleic acid are very different. At pH above the p*K*_a_, oleic acid is ionized, and it acts like a conventional ionic hydrophobic surfactant. However, at pH below the p*K*_a_, oleic acid is protonated, and its properties change to that of oil. Thus, oil droplets are formed when the oleic acid is mixed with water. For this reason, oleic acid can act as a dual oil and co-surfactant in the SNEDDS, improving ABZ solubility. Some excipients showed a relatively high solubilizing ability, as shown in Table 4. Based on these results, Tween 20, oleic acid, PEG 600 were selected for drug-loaded SNEDDS formulation development. Although we found some other excipients, such as PEG 300 and Cremophor EL, to show good solubility, we considered emulsification and drug loading parameters for the selection of surfactants and oils, and thus selected the above-listed excipients. SNEDDS are known to possess the ability to enhance solubility and absorption of the hydrophobic particles by increasing the net particle surface area and decreasing the size of the oil droplets, the latter of which are easily digestible and absorbed into mixed micelles that can easily traverse the intestinal lumen (Baloch et al., 2019). While formulating SNEDDS, it is important to avoid precipitation of the drug upon dilution in the gut lumen *in vivo*. Therefore, the components used in the formulation of SNEDDS should have increased solubility to ensure drug solubilization in the yielded dispersion.

**Table 4.** Solubility of ABZ in various oils, surfactants, and co-surfactants.

Vehicle	Total solubility (µg g^–1^)	Vehicle	Total solubility (µg g^–1^)
Sunflower oil	0.43	Labrasol	188.13
Captex 300	40.23	Cremophor RH40	46.72
Oleic acid	734.20	Acrysol K140	46.60
PEG 300	340.45	Maisine	18.43
PEG 600	498.90	Sesame oil	3.73
Soya oil	3.62	Capmul MCM	5.59
Olive oil	0.79	Capryol PGMC	179.48
Crodamol oil	23.61	Propylene glycol	194.47
Lauroglycol FCC	186.05	Captex 355	99.37
Cremophor EL	179.76	Tween 80	172.19
Solutol HS15	193.44	Acrysol K150	46.18
Tween 20	210.45	Capmul PG8	139.88
Labrafac lipophile WL1349	96.78	SPAN 20	185.79

### Construction of ternary phase diagram

**Figure 1.**
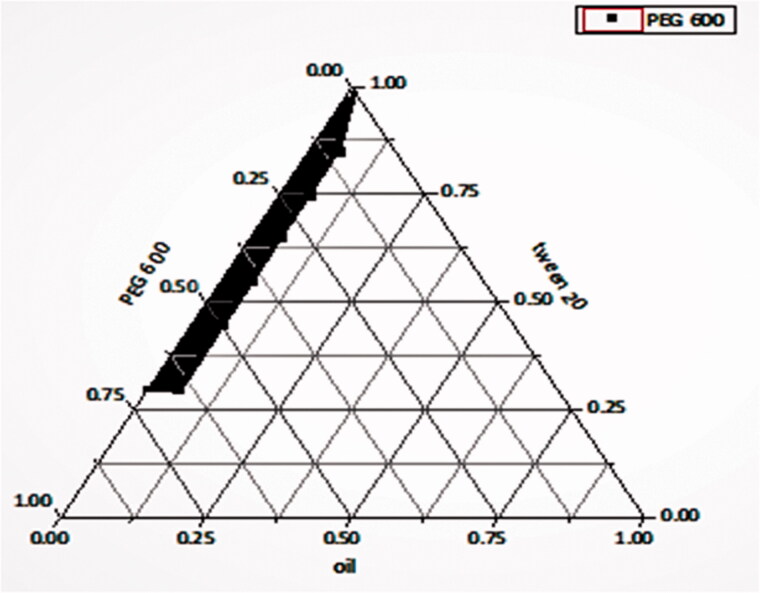
Ternary phase diagram showing the effect of oil (oleic acid), surfactant (tween 20), and co-surfactant (PEG 600). Nanoemulsion regions are shaded.

Figure 1 shows a ternary diagram of a phase system. Three species were used, and hence seven different systems were prepared at different concentrations using oleic acid as oil, tween 20 as surfactant, and PEG 600 as co-surfactant. The prepared systems were subsequently subjected to different characterization tests to select the best. The nanoemulsion regions are shaded. Pseudoternary phase diagrams were constructed to distinguish between self-nanoemulsifying regions and to select suitable concentrations of oil, surfactant, and co-surfactant for the formulation of SNEDDS.

### Characterization of the prepared SNEDDS

#### Globule size analysis

Globule size analysis is an important parameter used for characterization of successful SNEDDS system formulation. In terms of globule size analysis results (Table 5), it can be seen that the prepared system exhibits a mean globule size of <180 nm, which fulfills the criteria for a SNEDDS to have a mean globule size value <200 nm (Nasr et al., 2016). This is especially important, as the particle size is crucial to the drug dissolution rate and bioavailability, as reported in different studies (Chandra Sekhara Rao et al., 2004; Jinno et al., 2006; Ghosh et al., 2011). It is expected that SNEDDS in this size range offers an improved drug dissolution rate, subsequently resulting in increased bioavailability and thus facilitate reproducible time-dependent drug concentration profiles in the blood (Balakrishnan et al., 2009, 2013).

**Table 5.** Mean globule size of SNEDDS without drugs.

Formulation no.	Mean globule size (nm)±SD	PDI ± SD
1	173.6 ± 2.41	0.409 ± 0.024
2	115.6 ± 2.03	0.388 ± 0.021
3	16.4 ± 0.0765	0.394 ± 0.009
4	14.8 ± 0.620	0.450 ± 0.007
5	12.6 ± 0.563	2.265 ± 0.006
6	10.6 ± 0.662	0.480 ± 0.007
7	62.0 ± 1.05	0.240 ± 0.015

#### Determination of ABZ saturated solubility in the selected SNEDDS

As ABZ is a lipophilic drug with a log *p* value of 2.7, which is only slightly soluble in water, preparing a SNEDDS preconcentrate formulation enhances the drug solubility. The average solubility of ABZ in SNEDDS pre-concentrate was found to be 125 µg/mL, and the solubility in water was found to be 1.4 µg/mL, an average increase of 89-fold. This agrees with previous reports that SNEDDS possesses excellent potential for enhancing the oral bioavailability of highly lipophilic drugs (Borkar et al., 2016; Baloch et al., 2019). Thus, increased solubility of the SNEDDS formulation is expected to aid the bioavailability of ABZ following oral administration.

#### Preparation of supersaturated SNEDDS

A SNEDDS formulation that can maintain the loaded drug concentrations in amorphous form without the risk of being lost when dilution through precipitation is favored. A supersaturated solution is one with thermodynamically unstable property with high-energy drug molecules. When supersaturation is attained, the high-energy drug molecules in the preparation change to a lower energy level that is thermodynamically stable, by precipitation of its crystalline form. To benefit from this supersaturated state, high drug concentrations are needed to be maintained for longer periods so as to ensure enhanced drug absorption. We were able to achieve this through the addition of precipitation inhibitors. It has been previously reported that the action of precipitation inhibitors is through formation of hydrogen bonding with the drug molecules. These hydrogen bonds allow the precipitation inhibitor to adsorb onto the drug crystal surface, hindering the incorporation of more drug molecules within the crystal lattice, thus reducing the rate of growth of the crystal (Raghavan et al., 2001). Therefore, precipitation inhibitors slow down the precipitation of drug crystals on seed or drug nuclei, in a manner similar to how parachute slows down the downward motion of an object moving through the atmosphere. As the aim of this study was to prepare S-SNEDDS formulation, the selected SNEDDS was loaded with different drug concentrations (S-50% and S-100%) and different concentrations of HPMC E15 lv were used as precipitation inhibitors.

### Characterization of the prepared S-SNEDDS formulation

#### Globule size analysis

The mean globule size and mean PDI values of S-SNEDDS with or without HPMC E15 lv are shown in Table 6. No significant difference was observed in the formulated S-SNEDDS when compared with the SNEDDS formulation, as shown in Table 5. Similarly, no significant difference was observed for the mean globule size of the plain SNEDDS and that of the drug-loaded SNEDDS A or B (loaded with S-50% or S-100% in that order) with or without HPMC E15 lv. This similarity in the mean globule size values of both the SNEDDS and S-SNEDDS ensures that the loaded drug is encapsulated within the emulsion globule post-dilution.

**Table 6.** Mean globule size and pH of the S-SNEDDS and percentage transmittance of the different formulations.

S. no.	S-SNEDDS formulation no.	Mean globule size (nm)±SD	PDI ± SD	Mean pH ± SD	Percentage transmittance
1	A	58.9 ± 1.32	24.092 ± 0.02	6.8 ± 0.05	96
2	A1	78.4 ± 1.92	0.357 ± 0.004	6.8 ± 0.05	96
3	A2	101 ± 2.31	0.332 ± 0.002	6.8 ± 0.05	96
4	B	47.8 ± 1.02	11.741 ± 0.005	6.8 ± 0.07	97
5	B1	75.4 ± 1.53	2.647 ± 0.015	6.8 ± 0.07	97
6	B2	89 ± 1.96	0.278 ± 0.013	6.8 ± 0.07	100

#### Testing pH of S-SNEDDS

As the pH of a solution affects the phase separation, stability, and self-emulsifying nature of the S-SNEDDS, the pH levels of all preparations were measured using a pH meter (Table 6).

#### Turbidity measurement

Turbidity measurements were taken to identify the efficiency of self-emulsification by establishing whether the dispersion reached equilibrium rapidly and in a reproducible time. These measurements are commonly taken using UV visual spectroscopy, as in this study. Characterization of optical clarity was 100% (i.e. absorbance of suitably diluted aqueous dispersion at 248 nm). As shown in Table 6, the repeated measurements showed a narrow range of between 96 and 100% for the transmittance through the samples measured. As transmittance is a measure of how much of the transmitted light passes through the sample, it can be inferred here that the formulation had emulsified because the turbidity was very low (near zero due to transmittance).

#### SS-SNEDDS formulation study data

It has been found that the parameters of SS-SNEDD formulation range between 0.307 and 23. The parameters of bulk and tapped densities were found within the limit at 0.307 and 0.4, respectively. The Hausner ratio range was also excellent (at 1.30), with good flow (28.43) for the angle of repose. Finally, the data showed a poor compressibility index at 23 (see Table 7).

**Table 7.** SS-SNEDDS formulation study data.

Parameters	Range	Remarks
Bulk density	0.307	Within limits
Tapped density	0.4	Within limits
Hausner ratio	1.30	Excellent
Angle of repose	28.43	Good flow
Compressibility index	23	Poor

### Differential scanning calorimetry

**Figure 2.**
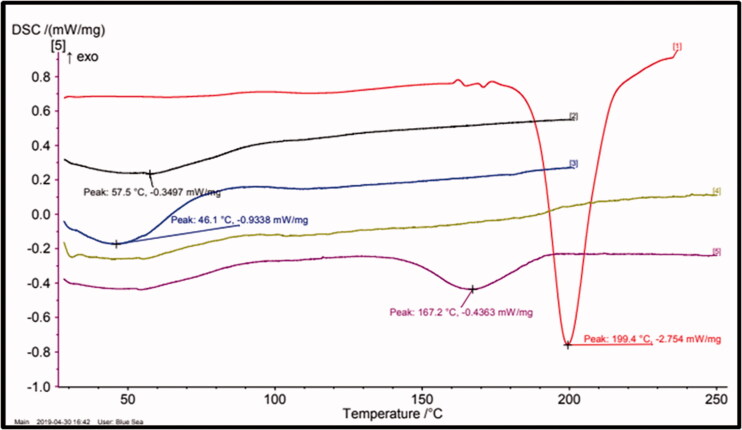
DSC heating curve of pure ABZ and SS-SNEDDS with different solid carriers (red line = drug, black line = SS-SNEDDS, blue line = HPMC, yellow line = MCC, purple line = physical mixture).

The DSC curve of pure ABZ and SS-SNEDDS formulation is shown in Figure 2. Pure ABZ showed a sharp melting endotherm at 199.4 °C. However, the endothermic peak of the ABZ was absent in the SS-SNEDDS formulation prepared with a solid carrier, which confirms the amorphous nature of ABZ in all SS-SNEDDS. Amorphous substances require less energy for their dissolution, resulting in increased dissolution rates, and thus, higher apparent solubility (Dash et al., 2015). This might explain the reason for the higher solubility of the SS-SNEDS observed in this study.

### Scanning electron microscopy

**Figure 3.**
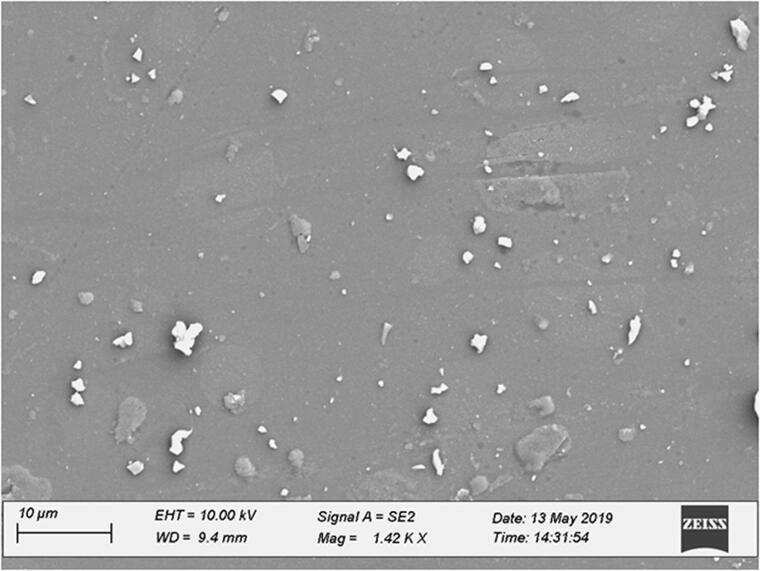
SEM of SS-SNEDDS prepared with microcrystalline cellulose.

The surface morphology and globule size of the formulated SNEDDS were also determined microscopically using SEM. A drop from the resultant nanoemulsion was diluted and examined. Figure 3 illustrates the SEM photograph of the solid carriers and SS-SNEDDS used to prepare the carriers. The SEM image shows spherical and homogenous droplets with a size smaller than 50 nm (Thoorens et al., 2014). The MCC is shown to have a microporous and crystalline structure, indicating incorporation of the drug inside the matrix system. It is clear that globules were well dispersed and no aggregation occurred. The MCC exhibited more than 90% intra-particle porosity when compared with the surface area. This suggests that the nominal particle size does not directly influence the particle surface area (Thoorens et al., 2014). In addition, as evident from the SEM, since the ABZ had been molecularly dissolved within the SS-SNEDDS matrix, the generated high surface area improved wettability and *in vitro* release of ABZ. The highly porous nature of the particle allows for swelling when in contact with an aqueous environment due to capillary action. Furthermore, this characteristic also favors disintegration for drug release due to the disruption of hydrogen bonds formed between water molecules and molecules of the particle shell.

## Conclusions and summary

In the present study, ABZ-loaded S-SNEDDS containing HPMC as a precipitation inhibitor were developed. Several SNEDDS were developed through the construction of ternary phase diagrams. The results of the investigation show that the optimized S-SNEDDS-B2 loaded with 10% (w/w) HPMC E15 lv was able to maintain super-saturation without precipitation. Thus, this offers an improved formulation for oral administration of ABZ that is hoped to improve both dissolution rate and bioavailability.

## Data Availability

The data used to support the findings of this study are available from the corresponding author upon request.
